# The Role of Diet in Women of Childbearing Age: Current Evidence Supporting Nutritional Recommendations

**DOI:** 10.3390/nu17223505

**Published:** 2025-11-09

**Authors:** Andrea Maugeri, Martina Barchitta, Giuliana Favara, Roberta Magnano San Lio, Claudia Ojeda-Granados, Elena Alonzo, Daniele Bellavia, Marialaura Bonaccio, Annalisa Di Nucci, Chiara Donfrancesco, Simona Esposito, Paolo Gandullia, Gianluca Giavaresi, Monica Giroli, Brunella Grigolo, Francesco Grassi, Francesco Leonardi, Elisa Proietti, Laura Sciacca, Licia Iacoviello, Antonella Agodi

**Affiliations:** 1 Department of Medical and Surgical Sciences and Advanced Technologies “GF Ingrassia”, University of Catania, 95123 Catania, Italy; andrea.maugeri@unict.it (A.M.); martina.barchitta@unict.it (M.B.); giuliana.favara@unict.it (G.F.); robertamagnanosanlio@unict.it (R.M.S.L.); claudiaojedagranados@hotmail.com (C.O.-G.); 2 Servizio Igiene degli Alimenti e Nutrizione (SIAN), Azienda Sanitaria Provinciale, 95100 Catania, Italy; 3 Istituto di Ricovero e Cura a Carattere Scientifico (IRCCS) Istituto Ortopedico Rizzoli, Scienze e Tecnologie Chirurgiche, 40136 Bologna, Italy; daniele.bellavia@ior.it (D.B.); gianluca.giavaresi@ior.it (G.G.); 4 Research Unit of Epidemiology and Prevention, Istituto di Ricovero e Cura a Carattere Scientifico (IRCCS) NEUROMED, 86077 Isernia, Italy; 5 Department of Cardiovascular, Endocrine-Metabolic Diseases and Aging, Istituto Superiore di Sanità, 00161 Rome, Italy; annalisa.dinucci@guest.iss.it (A.D.N.);; 6 Department of Medicine, University of Udine, 33100 Udine, Italy; 7 Department of Medicine and Surgery, Libera Università Mediterranea (LUM) University, 70010 Bari, Italy; 8 Unità Operativa Centrale (UOC) Gastroenterologia ed Endoscopia Digestiva, Istituto di Ricovero e Cura a Carattere Scientifico (IRCCS) G. Gaslini, 1647 Genoa, Italy; paologandullia@gaslini.org; 9 Centro Cardiologico Monzino Istituto di Ricovero e Cura a Carattere Scientifico (IRCCS), 20138 Milan, Italy; 10 Laboratorio RAMSES, Istituto di Ricovero e Cura a Carattere Scientifico (IRCCS) Istituto Ortopedico Rizzoli, 40136 Bologna, Italy; brunella.grigolo@ior.it (B.G.);; 11 Fondazione Italiana per l’Educazione Alimentare (FEI), 20214 Milan, Italy; 12 Department of Internal Medicine and Medical Specialties (DI.M.I.), University of Genoa, 16145 Genoa, Italy; elisa.proietti@edu.unige.it; 13 Nutritional Centre Istituto di Ricovero e Cura a Carattere Scientifico (IRCCS) G.Gaslini, 16147 Genoa, Italy; 14 Section of Endocrinology, Department of Clinical and Experimental Medicine, University of Catania, 16145 Catania, Italy

**Keywords:** nutrition, diet, childbearing age, nutritional recommendations

## Abstract

Background/Objectives: Nutrition during the reproductive years shapes women’s immediate health, fertility, pregnancy outcomes, and long-term offspring well-being. This position paper narratively synthesizes and critically appraises evidence on how dietary patterns, macro-/micronutrients, and supplementation influence women’s health, female fertility, and reproductive outcomes, to inform practical recommendations. Methods: We narratively reviewed recent reviews, cohort studies, clinical trials, and public-health guidance on macronutrients, key micronutrients, dietary patterns (with emphasis on the Mediterranean diet), ultra-processed food (UPF) intake, and targeted supplementation relevant to menstrual, metabolic, cardiovascular, skeletal, and reproductive outcomes. Results: Balanced, diverse diets rich in whole and minimally processed foods support hormonal regulation, ovulatory function, healthy gestation, and chronic-disease risk reduction. Priority nutrients include iron, folate, calcium, vitamin D, zinc, vitamin B12, and long-chain omega-3s (DHA), with supplementation considered when dietary intake or bioavailability is inadequate. Evidence consistently links Mediterranean-style eating to improved metabolic health, insulin sensitivity, IVF success, lower gestational diabetes risk, and favorable neonatal outcomes. High UPF consumption is associated with poorer diet quality, inflammation, adverse pregnancy outcomes, and potential reproductive impairment, warranting a reduction in favor of nutrient-dense foods. Diet also influences cardiovascular and bone health through effects on lipids, glycemia, blood pressure, and mineral/vitamin status, with fiber-rich carbohydrates, unsaturated fats (notably olive oil), and adequate calcium–vitamin D emerging as central levers. Conclusions: For women of childbearing age, a Mediterranean-aligned, minimally processed dietary pattern—tailored to individual needs and complemented by prudent use of folate, iron, vitamin D, calcium, B12, and DHA when indicated—offers robust benefits across reproductive, metabolic, cardiovascular, and skeletal domains. Public-health actions should improve access to healthy foods, curb UPF marketing, and embed personalized nutrition counseling in routine care; further longitudinal research from preconception through postpartum is needed.

## 1. Introduction

The role of nutrition in shaping the health and well-being of women of childbearing age is widely recognized as a cornerstone of both individual and public health. During this critical life stage, a woman’s nutritional status does not only influence her immediate health but also has profound effects on reproductive function, pregnancy outcomes, and the long-term development of future generations. Ensuring an adequate intake of essential nutrients, alongside adopting balanced dietary patterns, is therefore fundamental to supporting hormonal regulation, metabolic stability, and optimal reproductive outcomes.

Global dietary trends have undergone significant transformations, often marked by a progressive shift away from traditional, nutrient-rich diets and an increased reliance on ultra-processed foods. This shift raises important concerns, as emerging evidence suggests a strong relationship between dietary quality and a range of health outcomes in women of reproductive age. Against this backdrop, the present position paper aims to provide an up-to-date and comprehensive synthesis of current scientific knowledge on the role of diet in this population, with the goal of offering evidence-based nutritional recommendations tailored to their specific physiological needs. This position paper was developed within Trajectory 5 (“Nutraceuticals, Nutrigenomics, and Functional Foods”) and Action 5.1 (“Creation of an Action Plan to Combat Malnutrition in All Its Forms and to Promote the Principles of the Mediterranean Diet”), under the Health Operational Plan—Development and Cohesion Fund 2014–2020, funded by the Italian Ministry of Health. For each topic, a domain expert identified and shortlisted the most relevant and recent evidence using pre-specified priorities (women of childbearing age; prospective designs; systematic reviews/meta-analyses; randomized trials when available). The shortlist was then discussed by the full author group; disagreements were resolved by consensus, and the final selection and interpretation were approved by all co-authors. Our aim is to provide healthcare professionals, policymakers, and women of childbearing age with a clear, evidence-informed framework to improve diet quality across the reproductive years, translating current research into practical recommendations that support women’s health, fertility, and the long-term well-being of future generations.

## 2. Nutritional Recommendations for Women of Childbearing Age

### 2.1. Importance of Nutritional Balance and Dietary Variety

The health of women of childbearing age is foundational to their own well-being and to the healthy development of future generations. Nutritional requirements during this life stage are unique and demanding, encompassing not just the need for general well-being but also preparation for pregnancy, healthy fetal development, and lactation [1]. This chapter explores the importance of balanced nutrition for women of childbearing age and provides detailed guidance on macronutrient, micronutrient, and supplement requirements.

A balanced and diverse diet is essential for maintaining optimal health, supporting reproductive functions, and preparing for the challenges of pregnancy [2]. Nutritional balance ensures that women receive the broad range of nutrients needed to support the body’s various functions, while dietary variety is a key indicator of diet quality [3]. According to a review on dietary diversity among women of reproductive age in low- and middle-income countries (LMICs), it was found that the majority of these women consumed poorly diversified diets, often lacking key micronutrients like calcium, iron, and vitamin A [4].

The Minimum Dietary Diversity for Women (MDD-W) is an essential metric, suggesting that consuming at least five different food groups per day can significantly improve the likelihood of meeting micronutrient needs [5]. However, data suggests that many women do not meet this diversity threshold, especially in regions experiencing food insecurity. Inadequate dietary diversity is linked to adverse reproductive health outcomes, including complications during pregnancy and long-term developmental issues in offspring [6]. Furthermore, studies like the ELANS Study have highlighted that women in Latin America who achieve a diverse diet (MDD-W > 5) have a significantly higher intake of most micronutrients, although they still often fall short in meeting requirements for vitamins D and E [7].

Improving dietary diversity can be challenging, particularly in low-resource settings. However, incorporating the following strategies can significantly help: (i) community education programs could increase awareness about the importance of including various food groups in daily meals, by providing nutritional information and encouraging women to make better food choices; home gardening initiatives are especially useful in low-income communities where fresh produce might be expensive or unavailable; improving access to fresh foods by connecting local farmers with communities, which ensure seasonal fruits and vegetables are accessible; in regions where certain micronutrients are consistently lacking, promoting fortified foods can be beneficial [8,9,10].

### 2.2. Essential Macronutrients: Proteins, Carbohydrates, and Fats

Macronutrients—proteins, carbohydrates, and fats—are the building blocks of a healthy diet, playing a unique and crucial role in supporting reproductive health and general well-being [11]. Proteins are vital for cell growth and repair, and during pregnancy, they are particularly important for the development of the fetus and placenta. Ensuring an adequate intake of proteins also supports the body’s immune system and maintains muscle mass, which is crucial during pregnancy [12]. Sufficient protein intake has been associated with healthier birth weights and better preservation of maternal muscle mass after delivery. Protein requirements increase substantially during pregnancy to support fetal development and maternal health. It is recommended that pregnant women consume at least 1.1 g of protein per kilogram of body weight [13]. These needs rise progressively throughout pregnancy, with an estimated additional 1 g per day in the first trimester, 9 g per day in the second, and 29 g per day in the third trimester [14].

Incorporating a variety of protein-rich foods such as lean meats, fish, eggs, dairy products, legumes, soy, lentils, chickpeas and beans, nuts is recommended for meeting daily protein needs and can provide a complete amino acid profile. This diversity is especially critical for vegetarian and vegan women to ensure they meet their nutritional needs effectively [15].

Carbohydrates are the body’s primary energy source, essential for daily activities and meeting the increased energy needs of pregnancy. Choosing complex carbohydrates from whole grains, vegetables, and fruits ensures a consistent energy supply while helping to maintain stable blood sugar levels. In contrast, simple sugars should be minimized to reduce the risk of gestational diabetes and other complications associated with blood sugar fluctuations [16].

Foods like oats, brown rice, quinoa, and whole wheat are excellent sources of complex carbohydrates that also provide dietary fiber. Fiber plays a vital role in supporting digestive health during pregnancy, helping to prevent constipation, a common concern for expectant mothers. Prioritizing fiber-rich carbohydrate sources is key to promoting gastrointestinal well-being and ensuring optimal health throughout pregnancy [17].

Fats play a crucial role in the overall health of women of childbearing age, supporting hormonal balance, the absorption of fat-soluble vitamins (A, D, E, K), and overall well-being. The primary fat source in the Mediterranean diet is olive oil, rich in monounsaturated fatty acids, especially oleic acid, which constitutes 70–80% of its composition, and bioactive polyphenols. In addition, healthy fats, particularly n-3 PUFAs, offer significant benefits, including reducing inflammation and supporting brain health. Sources like fish, flaxseed, walnuts, and chia seeds provide these essential nutrients, making them valuable components of a balanced diet [18].

For women planning a pregnancy, n-3 PUFAs, such as docosahexaenoic acid (C22:6n-3, DHA), are particularly important for supporting the development of a healthy nervous system in future offspring. Experts recommend consuming at least 200–300 mg of DHA daily during preconception, pregnancy, and lactation [19]. Fatty fish, such as salmon and sardines, are excellent dietary sources, and women are encouraged to include them in their meals two to three times per week [20]. Additionally, the balance between n-3 and n-6 PUFAs is important for reducing inflammation and promoting hormonal health. While omega-6 fats, found in vegetable oils like sunflower and soybean oil, are necessary in moderation, excessive intake can be pro-inflammatory. Ensuring an adequate intake of n-3 PUFAs helps maintain this balance, supporting not only reproductive health but also long-term vitality for women of childbearing age [21].

### 2.3. Key Micronutrients: Vitamins and Minerals

Women of childbearing age have increased requirements for certain vitamins and minerals due to the physiological demands of menstruation, pregnancy, and lactation. Ensuring sufficient intake of these micronutrients is critical for both maternal health and positive pregnancy outcomes [22].

Iron is important for preventing anemia, a common condition among women of childbearing age, and for meeting the physiological demands of menstruation and other factors. Key dietary sources of iron include lean red meats, legumes, and leafy green vegetables. Vitamin C enhances iron absorption and should be included with iron-rich meals. It is important to monitor iron levels, as deficiency can lead to fatigue, weakness, and other symptoms related to low oxygen availability in the body [23]. Women of reproductive age should aim for approximately 18 mg of iron per day [23], compared to the higher 27 mg recommended during pregnancy [24]. This increased need may require supplementation, particularly in cases of deficiency or when dietary absorption is insufficient. Iron absorption can be inhibited by certain dietary components, such as phytates, calcium, and tannins. To maximize iron absorption, it is advisable to separate the consumption of iron supplements or iron-rich meals from foods containing these inhibitors. Additionally, including a source of vitamin C with iron-rich meals can boost absorption by up to four times [25].

Folate (vitamin B9) is important for women of childbearing age, as it plays a critical role in preventing neural tube defects in a developing fetus. It is recommended that all women who are capable of conceiving consume 400 mcg of folic acid daily, either through fortified foods or supplements. Natural sources of folate include leafy greens, legumes, and citrus fruits, although supplementation is often necessary to meet the body’s needs [26].

While the recommended intake of folate increases during pregnancy, maintaining adequate levels before conception is equally important for overall health. Folate is vital for DNA synthesis, cell division, and proper reproductive function. It also supports epigenetic modifications that affect gene expression without changing the DNA sequence. Ensuring sufficient folate intake during the childbearing years has been linked to improved reproductive health, better fertility, and long-term health benefits, including reduced risks of developmental delays in offspring and better metabolic and cardiovascular health later in life [5].

Calcium is crucial for maintaining bone health in women of childbearing age and for supporting proper bone development during pregnancy. Many women, however, do not meet their daily calcium requirements, which can increase their risk of osteoporosis later in life. Excellent dietary sources of calcium include dairy products, fortified plant-based milks, leafy greens and calcium-rich mineral water [27]. Vitamin D is also crucial for the absorption of calcium, and a deficiency in either calcium or vitamin D can negatively impact bone health for both the mother and future child. Ensuring adequate calcium intake during the childbearing years is important not only for bone strength but also for overall health. Although the calcium needs increase during pregnancy and lactation (around 1200 mg/day), maintaining sufficient levels before conception is important for long-term bone health and overall well-being [28]. If dietary sources are insufficient, calcium supplements can help bridge the gap. Calcium competes with iron for absorption. Therefore, it is beneficial to space out calcium and iron supplements. Including magnesium-rich foods such as avocado, nuts, and dark chocolate can also help with calcium absorption and prevent muscle cramps during pregnancy [29].

Vitamin D works in tandem with calcium to support bone health and overall well-being in women of childbearing age. A deficiency in vitamin D is common among women, particularly those with limited sun exposure. In such cases, supplementation may be necessary to ensure adequate levels. Beyond its role in bone health, sufficient vitamin D is also linked to a reduced risk of preeclampsia, a serious pregnancy complication. Ensuring adequate vitamin D intake during the childbearing years is important not only for maintaining strong bones but also for supporting reproductive health and reducing the risk of complications in future pregnancies [30]. Women should aim for 600 IU of vitamin D daily, and those with minimal sun exposure might require even higher doses, often guided by healthcare professionals through regular monitoring of blood levels [31]. Besides its role in bone health, Vitamin D plays an important role in immune modulation. During pregnancy, Vitamin D can help reduce the risk of infections and improve immune response, which is crucial for maternal and fetal health [5].

Zinc and vitamin B12 are essential for reproductive health in women of childbearing age and play key roles in overall well-being. Zinc is important for immune function, cell division, and DNA synthesis, while vitamin B12 supports proper neurological function and red blood cell formation. Women are advised to consume around 11 mg of zinc daily, especially during pregnancy, to support enzyme activity essential for cell growth and DNA synthesis. Vitamin B12, found primarily in animal products, should be consumed at 2.6 mcg per day during pregnancy to promote healthy fetal neural development and maternal health [32]. For women following vegetarian or vegan diets, ensuring adequate intake of vitamin B12 is especially important, as they are at a higher risk of deficiency. Fortified foods and supplements can help meet B12 requirements and prevent anemia and neurological complications, supporting both reproductive health and overall vitality during the childbearing years [33].

Iodine is important for the synthesis of thyroid hormones, which are critical for fetal brain development, growth, and metabolic regulation. Inadequate iodine intake during pregnancy can impair neurodevelopment, leading to lower intelligence quotient, developmental delays, and in severe cases, cretinism. A woman’s iodine requirements increase substantially during pregnancy to ensure an adequate supply to the fetus and support the mother’s thyroid function. The recommended daily intake of iodine during pregnancy ranges from 200 to 250 mcg.

Most commonly consumed foods are relatively low in iodine content, and dietary intake alone is often insufficient to meet increased demands during pregnancy and lactation. Key dietary sources include seafood, dairy products, eggs, and iodized salt. However, in many regions, universal salt iodization (USI)—the WHO and UNICEF-recommended public health strategy to prevent iodine deficiency—is not consistently implemented. Ensuring sufficient iodine intake during the childbearing years is crucial not only for maternal thyroid function but also for optimal fetal and early childhood cognitive development. In regions without reliable iodized salt coverage, supplementation is a critical intervention to bridge this nutritional gap and prevent adverse health outcomes [5,34].

### 2.4. Role of Dietary Supplements in Supporting Reproductive Health

Dietary supplements are often necessary to fill nutritional gaps, particularly for women who may not meet their nutrient needs through diet alone. Studies have highlighted the efficacy of supplements in reducing nutrient deficiencies, particularly for folate, iron, vitamin D, and n-3 PUFAs. These nutrients are critical for optimizing reproductive health, preventing deficiencies that could lead to adverse pregnancy outcomes, and supporting lactation [35]. Folate supplementation is especially important for women of childbearing age and should be considered before conception to help prevent neural tube defects. It is recommended that women begin supplementation of 400–800 mcg of folic acid at least one month prior to conception and continue through the first trimester. For women with a history of neural tube defects or certain genetic conditions that affect folate metabolism, higher doses may be advised [36].

Iron supplementation may also be necessary for women at risk of anemia, particularly those with low dietary iron intake or increased iron requirements. Iron is crucial for preventing fatigue, supporting immune function, and ensuring healthy fetal development. Adequate iron levels before and during pregnancy help reduce the risk of anemia and promote overall reproductive health [5].

Pregnant women are typically advised to take an iron supplement of 30–60 mg daily, depending on their hemoglobin levels, to prevent iron deficiency anemia. While supplementation is essential for maintaining adequate iron levels, excessive intake can lead to toxicity, which may cause symptoms such as constipation, nausea, and even oxidative damage. To avoid the risks associated with iron overload, regular monitoring of iron status through blood tests is recommended to ensure optimal levels and prevent complications [37]. Vitamin D and calcium supplementation can be beneficial for women of childbearing age, especially those with limited sun exposure or insufficient dietary intake. Both nutrients are essential for bone health and overall reproductive function. Vitamin D deficiency is also linked to complications like gestational diabetes and preeclampsia, highlighting the need for regular monitoring of vitamin D levels. For women whose dietary calcium intake is insufficient, supplements of 500 mg twice daily are recommended, particularly for those with low dairy consumption [38].

n-3 PUFAs, especially DHA, are crucial for brain and eye development, making them important not just during pregnancy but also for women in their childbearing years who are planning to conceive. Fish oil supplements or algae-based n-3 PUFAs are often recommended, particularly for women who do not consume fish regularly. These supplements support the development of the fetal nervous system and have been linked to a reduced risk of preterm birth. A daily intake of at least 200 mg of DHA is recommended for pregnant and lactating women. For vegetarians, algae-based supplements offer a suitable alternative. While flaxseeds and chia seeds are rich in alpha-linolenic acid (ALA), which can be converted to DHA, this conversion is inefficient in humans, making supplementation a more effective option [39].

Healthcare providers play a key role in assessing the nutritional needs of women and recommending appropriate supplements based on their individual health status and dietary habits. It is also essential for healthcare providers to educate women about the potential risks of excessive supplementation, such as vitamin A toxicity, which can lead to congenital disabilities. Proper guidance and monitoring are crucial for maintaining balanced nutrition and ensuring overall reproductive health [40].

## 3. Impact of Dietary Patterns

### 3.1. Mediterranean Diet and Its Benefits

Nutrition during the childbearing years is critical for women’s health and the quality of future pregnancies. In this chapter, we explore the role of different dietary patterns, focusing particularly on the Mediterranean Diet, comparing it with other dietary models, and discussing the importance of preconception nutrition. Recent reviews highlight the relationship between dietary habits, metabolic health, and reproductive outcomes [41,42].

The Mediterranean Diet is consistently recognized as one of the healthiest dietary patterns, offering numerous benefits for women of childbearing age. Characterized by high consumption of fruits, vegetables, legumes, whole grains, nuts, and extra virgin olive oil, with moderate intake of fish, dairy products, and poultry, and limited consumption of red meat and processed foods, the Mediterranean Diet is particularly advantageous for reproductive health.

Recent studies indicate that the Mediterranean Diet provides significant benefits due to its anti-inflammatory and nutrient-dense properties. Silvestris et al. report that the diet’s rich content of antioxidants, n-3 PUFAs, and healthy fats contributes to improving oocyte quality and reducing systemic inflammation, which are crucial for fertility [41]. Antioxidants, such as vitamins C and E, coenzyme Q10, and glutathione, play an essential role in combating oxidative stress—a key factor that can impair reproductive function by damaging oocytes and disrupting hormonal balance [41].

A study by Vujkovic et al. also highlighted that the Mediterranean Diet was associated with improved in vitro fertilization (IVF) success rates, emphasizing the impact of a balanced, antioxidant-rich diet on reproductive outcomes [43]. These benefits are further supported by evidence showing that n-3 PUFAs, particularly those found in fatty fish, improve endometrial receptivity, which enhances the likelihood of embryo implantation [44]. These findings suggested that the Mediterranean Diet plays a role in supporting both natural conception and assisted reproductive techniques.

Grieger et al. also emphasize that adopting a Mediterranean-style dietary pattern was associated with improved preconception health and a reduction in gestational diabetes risks. This evidence highlights the broader benefits of the Mediterranean Diet beyond fertility, supporting long-term health for both mothers and their offspring [45].

In addition, the Mediterranean Diet was associated with enhanced insulin sensitivity, which is particularly beneficial for women suffering from polycystic ovary syndrome (PCOS) or obesity-related infertility. Insulin resistance, which is prevalent in women with PCOS and those who are overweight, can negatively affect ovarian function by promoting hyperinsulinemia. This condition impairs oocyte maturation and disrupts ovulatory cycles. By emphasizing whole grains, fiber, unsaturated fats, and n-3 PUFAs, the Mediterranean Diet helps regulate blood sugar levels and improve insulin sensitivity, leading to better ovulatory function [41].

n-3 PUFAs, particularly those found in fatty fish such as salmon, sardines, and mackerel, are key components of the Mediterranean Diet. These fats provide anti-inflammatory benefits, improve blood flow to reproductive organs, and help maintain a balanced hormonal environment. n-3 PUFAs also regulate prostaglandin production, which is important for ovulation, menstrual regulation, and embryo implantation [41].

A meta-analysis by Fabiani et al. concluded that adherence to the Mediterranean Diet significantly reduced the risk of metabolic syndrome, which is often linked to infertility due to hormonal imbalances. The combination of anti-inflammatory components, antioxidants, and n-3 PUFAs in the Mediterranean Diet fosters a holistic approach to maintaining reproductive health and improving pregnancy outcomes [46].

The evidence from 33 studies, including randomized controlled trials and observational analyses, consistently highlights the beneficial role of the Mediterranean Diet in pregnancy. Adherence to the Mediterranean Diet was associated with a significant reduction in the incidence of gestational diabetes when compared with both standard and low-fat dietary approaches. Importantly, randomized trials demonstrated a high level of certainty for this outcome, showing a meaningful reduction in risk. The Mediterranean Diet also proved effective in lowering the likelihood of adverse neonatal outcomes, including both low and high birth weight, with strong certainty of evidence. Furthermore, moderate certainty evidence suggested that maternal infections are less frequent among women following a Mediterranean-style dietary pattern. Taken together, the findings support recommending the Mediterranean Diet as a safe and effective nutritional approach during pregnancy, with substantial benefits for both maternal and neonatal health [47].

### 3.2. Comparison with Other Dietary Patterns

When comparing the Mediterranean Diet with other popular dietary patterns, such as the Western diet and vegetarian/vegan diets, distinct differences emerge regarding their effects on reproductive health.

The Western diet, typically characterized by high intakes of processed foods, refined sugars, and saturated fats, has been linked to adverse health outcomes that can severely impact fertility. Obesity and insulin resistance, which are often associated with the Western diet, contribute significantly to ovulatory dysfunction and impaired oocyte quality. According to Silvestris et al., the high consumption of refined carbohydrates and saturated fats common in the Western diet leads to chronic inflammation and metabolic disturbances, disrupting the delicate hormonal balance necessary for conception [41].

Furthermore, a study by Rich-Edwards et al. [48] found that women consuming a Western diet were more likely to experience issues related to anovulation and hormonal imbalance, indicating that such a diet could have long-term consequences for reproductive health. In contrast, dietary patterns that include more whole foods, antioxidants, and unsaturated fats, like the Mediterranean Diet, demonstrate a much more favorable impact on fertility [48].

Grieger et al. [45] also discusses the importance of minimizing intake of fast food and sugar-sweetened beverages before conception. High intakes of these foods have been linked to longer times to pregnancy and reduced fertility, suggesting that dietary interventions focused on reducing processed foods and sugars can improve reproductive outcomes [45].

In contrast, vegetarian and vegan diets, which focus on plant-based foods, offer many health benefits, including reduced risks of obesity and improved cardiovascular health. However, vegetarian diets require careful planning to ensure that essential nutrients, such as protein, vitamin B12, iron, zinc, and n-3 PUFAs, are adequately provided. Deficiencies in these nutrients can adversely affect ovulatory function and overall fertility. The Mediterranean Diet offers a more balanced approach, including both plant-based foods and moderate animal protein sources, ensuring a comprehensive nutrient profile that supports optimal reproductive health [41].

The Nurses’ Health Study II found that dietary patterns low in trans fats and refined sugars and rich in monounsaturated fats, plant proteins, and low-glycemic carbohydrates—similar to the Mediterranean Diet—were associated with a significantly reduced risk of ovulatory infertility. This evidence underscores the importance of dietary choices that support metabolic health, reduce inflammation, and maintain hormonal balance, all of which are essential for reproductive success [49].

### 3.3. Importance of Preconception Nutrition

Preconception nutrition plays a pivotal role in preparing a woman’s body for pregnancy and enhancing fertility outcomes. Proper nutrition during the preconception phase ensures that a woman’s body has sufficient reserves of critical nutrients required for conception and the early stages of fetal development. However, different dietary patterns may provide these nutrients in varying quantities and bioavailability, impacting their effectiveness in supporting reproductive health.

The Mediterranean Diet’s emphasis on nutrient-dense foods provides many of the essential vitamins and minerals necessary for reproductive health, including folate, iron, calcium, and n-3 PUFAs. This diet ensures a diverse and balanced intake of these key nutrients, which are critical during the preconception phase. However, other dietary patterns, such as vegetarian or vegan diets, may require additional planning or supplementation to achieve comparable nutrient levels.

Folate is a vital nutrient during the preconception period. It plays a critical role in DNA synthesis, repair, and methylation, which are especially important during early fetal development. Adequate folate intake before conception helps reduce the risk of neural tube defects and supports healthy cell division. Folate also supports the 1-Carbon Cycle, which is essential for maintaining genetic stability during conception and embryonic development [50]. The Mediterranean Diet’s rich inclusion of leafy green vegetables, legumes, and nuts makes it an excellent source of natural folate; vegetarian and vegan diets also provide folate through plant-based sources, but the bioavailability may vary, often supplementation may be necessary to meet recommended levels.

Iron is another key nutrient for women of childbearing age, particularly during the preconception period. Iron deficiency is one of the most common nutritional deficiencies in women worldwide and can negatively impact ovulatory function. The Mediterranean Diet includes various iron-rich foods, such as legumes, leafy greens, lean meats, and fish, which provide heme and non-heme iron. Heme iron, found in animal products, is more readily absorbed by the body compared to non-heme iron, which is prevalent in plant-based diets. Vegetarian and vegan diets may require careful planning to ensure adequate iron intake and often need to incorporate vitamin C-rich foods to enhance non-heme iron absorption. Adequate iron levels reduce the risk of anemia, which can lead to fatigue and pregnancy complications, including preterm birth and low birth weight [51].

n-3 PUFAs are also crucial during the preconception phase, as they help reduce inflammation, support hormone production, and enhance blood flow to reproductive organs. n-3 PUFAs, especially those found in fish, flaxseeds, and walnuts, are known to improve egg quality and create a favorable environment for implantation. The Mediterranean Diet, with its emphasis on fatty fish, provides an excellent source of long-chain n-3 PUFAs (eicosapentaenoic acid (C20:5n-3, EPA) and DHA), which are particularly beneficial for reproductive health. In vegetarian and vegan diets, n-3 PUFAs are primarily derived from plant-based sources like flaxseeds and walnuts, which provide alpha-linolenic acid (ALA). The body must convert ALA to EPA and DHA, but this conversion is often inefficient, necessitating supplementation in some cases to ensure optimal n-3 PUFA levels [44].

Antioxidants are vital for reproductive health as they protect against oxidative stress, which can damage both oocytes and sperm. Oxidative stress arises when there is an imbalance between free radical production and the body’s ability to neutralize them using antioxidants. This imbalance can impair oocyte quality and hinder embryo development. The Mediterranean Diet, rich in antioxidants from fruits, vegetables, nuts, and olive oil, helps maintain oxidative balance, which is crucial for reproductive success. Vegetarian and vegan diets can also provide a high intake of antioxidants due to their plant-based nature, though the variety and concentration may differ depending on dietary diversity and choices. Studies have shown that women who consume diets high in antioxidants have higher conception rates and lower risks of pregnancy complications [52].

Grieger et al. [45] also points out that improving preconception nutrition, particularly with an overall healthy diet or a Mediterranean-style dietary pattern, can help reduce the risks of conditions like gestational diabetes. Ensuring adequate micronutrient intake before conception can positively influence maternal and child health outcomes throughout pregnancy [45].

For all these reasons, the impact of dietary patterns on women of childbearing age is profound, with the Mediterranean Diet emerging as a particularly beneficial model for optimizing reproductive health. Compared to other dietary patterns, such as the Western diet and vegetarian/vegan diets, the Mediterranean Diet provides a balanced approach that includes essential nutrients, healthy fats, and antioxidants—all of which collectively support ovarian health, insulin sensitivity, and overall fertility.

Preconception nutrition plays a pivotal role in enhancing a woman’s ability to conceive and sustain a healthy pregnancy. The Mediterranean Diet, with its focus on nutrient-dense, anti-inflammatory foods, provides key nutrients such as folate, iron, n-3 PUFAs, and antioxidants, all of which are essential for reproductive success. However, other dietary patterns may vary in their ability to provide these nutrients, and careful planning or supplementation may be necessary to achieve optimal levels. Adopting a balanced dietary pattern like the Mediterranean Diet can help women not only improve their chances of conceiving but also lay a strong foundation for a healthy pregnancy and a healthy baby. The current evidence highlights the importance of adopting a Mediterranean-inspired dietary pattern that emphasizes whole, unprocessed foods, healthy fats, and balanced nutrient intake. By focusing on preconception nutrition and choosing a diet that supports metabolic and reproductive health, women of childbearing age can significantly enhance their fertility outcomes and overall well-being.

## 4. The Potential Role of Ultra-Processed Foods

From a public-health perspective, ultra-processed foods (UPFs) contribute a substantial share of dietary energy in many populations and are typically energy-dense and nutrient-poor, characterized by refined carbohydrates, added sugars, saturated and trans fats, and high sodium, often combined with cosmetic additives that enhance palatability and shelf-life. The growing consumption of UPFs has become a topic of increasing concern due to its potential impact on human health [53,54], particularly in women of childbearing age. This trend has paralleled a global decline in adherence to traditional dietary patterns, such as the Mediterranean diet [55]. According to the NOVA system, UPFs are industrially formulated products characterized by the presence of artificial ingredients, additives, preservatives, and flavor enhancers, and are often composed of substances extracted or derived from foods rather than whole ingredients [56,57]. These products are specifically engineered to be hyper-palatable, have extended shelf lives, and encourage overconsumption [58]. From a nutritional standpoint, UPFs tend to be energy-dense yet nutrient-poor, typically high in saturated and trans fats, added sugars, and salt, while offering minimal amounts of essential nutrients [59]. Several studies have shown that individuals whose diets are largely composed of UPFs tend to exhibit poorer overall diet quality, including lower adherence to health-promoting dietary patterns such as the Mediterranean diet [60].

The introduction of the NOVA classification [56,57] has marked a shift in nutrition science, moving beyond the traditional focus on individual nutrients to consider the degree of food processing as a determinant of health. This approach has contributed to broadening our understanding of dietary patterns and health outcomes. However, it is important to recognize that NOVA, like other dietary classification systems and scores [61,62], remains the subject of scientific debate [63]. For instance, it has been argued that the health risks attributed to UPFs may not derive solely from the degree of processing itself [63], and that the subjective nature of food processing classifications and the inconsistency among various systems might complicate a clear interpretation of the data [64].

Despite these discussions, numerous observational epidemiological studies consistently report associations between higher UPF consumption and increased risk of mortality, chronic diseases, and overall poor diet quality, often linked to reduced adherence to healthier dietary models such as the Mediterranean diet [53,60].

Specifically concerning women’s health during the fertile period, some studies suggested that a high intake of UPFs during pregnancy may be linked to negative outcomes, including gestational diabetes, poor glycemic control, excessive weight gain, inflammation, and hypertensive disorders [65,66]. There is also evidence indicating potential adverse effects on child growth and development, contributing to adiposity, mental health issues, and poor diet quality in offspring [66]. Furthermore, the pro-inflammatory potential of UPFs—linked to both their nutritional composition and the presence of food additives, contaminants, and packaging materials—has raised concerns about their impact on female reproductive health. Elevated levels of inflammatory markers like C-reactive protein and pro-inflammatory cytokines observed with high UPF consumption [67] may influence critical reproductive processes, including oocyte quality, folliculogenesis, hormone production, and immune signaling, ultimately affecting fertility [68]. Moreover, chronic maternal inflammation during pregnancy has been associated with neurodevelopmental disorders in offspring [69].

Additional concerns include the presence of advanced glycation end products (AGEs) in many UPFs, particularly in fried and baked goods. AGEs are implicated in reproductive and metabolic dysfunctions associated with polycystic ovary syndrome (PCOS), one of the leading causes of infertility in women [70,71]. UPFs also commonly involve plastic packaging, contributing to exposure to synthetic chemicals such as phthalates and bisphenols—substances recognized as endocrine-disrupting chemicals (EDCs). EDC exposure has been linked to irregular reproductive cycles, premature ovarian insufficiency, and diminished ovarian reserve [72,73]. Collectively, these pathways provide biologic plausibility linking UPF intake to impaired female fertility and—despite incomplete causal evidence—support a precautionary recommendation to limit UPFs among women planning pregnancy. While there is credible concern about the overconsumption of UPFs and their potential role in reducing overall diet quality and increasing exposure to undesirable compounds, it is crucial to adopt a balanced perspective. Promoting dietary patterns based on fresh, minimally processed foods rich in nutrients, such as fruits, vegetables, legumes, and whole grains, remains a sound strategy to support women’s health during their reproductive years. At the same time, caution is warranted before attributing adverse health outcomes exclusively to the level of food processing without further robust, causal evidence. Ultimately, focusing on overall diet quality—rather than processing alone—may represent the most pragmatic and scientifically grounded approach.

## 5. Dietary Implications for General Health

### 5.1. Physiological Factors Influenced by Diet

Diet profoundly influences physiological factors in women, affecting hormonal regulation, reproductive health, metabolism, and overall well-being [74]. Nutrient-dense diets, such as the Mediterranean diet or plant-based diets, rich in monounsaturated fats, antioxidants, and n-3 PUFAs, enhance hormonal stability by reducing systemic inflammation and supporting estrogen and progesterone balance and lower risks of metabolic syndrome [75]. This dietary pattern has been linked to improved reproductive health and lower risks of conditions like PCOS [75]. Essential nutrients like selenium and iodine support thyroid function. Conversely, diets high in saturated fats and refined sugars can disrupt hormonal equilibrium, contributing to insulin resistance, obesity, and menstrual irregularities [74]. Elevated insulin levels, often associated with high-fat diets, exacerbate androgen production, worsening symptoms of hormonal disorders like PCOS. Leptin and ghrelin, hormones regulating appetite and energy balance, are also influenced by diet [76]. Poor dietary habits, such as frequent consumption of high-calorie, nutrient-poor foods, alter leptin sensitivity, increasing hunger and promoting weight gain, which further disrupts hormonal function [76]. Tailored dietary strategies focusing on whole foods, fiber, and healthy fats can help stabilize hormonal fluctuations, optimize reproductive health, and reduce the risks of endocrine-related conditions [77]. Additionally, women’s unique body composition and hormonal fluctuations emphasize the need for gender-specific dietary strategies to optimize hormonal balance, weight management and cardiovascular function [74].

### 5.2. Impact on Obesity and Weight Management

Dietary quality, along with eating habits and lifestyle factors, plays a key role in the development of obesity and weight control among women. Excessive consumption of calorie-dense, nutrient-poor foods (e.g., sugary beverages, ultra-processed snacks, and fast foods) leads to a sustained positive energy balance, promoting adipose tissue accumulation [78,79].

In individuals affected by metabolic conditions such as type 2 diabetes, metabolic syndrome, obesity, or non-alcoholic fatty liver disease, the Mediterranean Diet emerges as a valuable dietary strategy. Evidence from cohort studies suggested that adherence to the Mediterranean Diet is linked with reduced mortality, particularly among patients with diabetes and metabolic syndrome. Clinical trials further indicate improvements in key metabolic outcomes, including reductions in body mass index, waist circumference, and body weight in specific patient groups. Lipid profiles also benefited: Mediterranean Diet lowered total and LDL cholesterol, increased HDL cholesterol, and reduced triglyceride concentrations, with positive effects observed both in adults and in pediatric populations with obesity. Glycemic control was significantly improved, with reductions in fasting glucose and HbA1c, alongside decreased insulin resistance measured by HOMA-IR. Inflammatory status, as indicated by serum C-reactive protein, also improved under Mediterranean Diet adherence. Notably, in patients with metabolic dysfunction-associated steatotic liver (MASLD; formerly nonalcoholic fatty liver disease, NAFLD), the Mediterranean Diet was associated not only with metabolic improvements but also with an enhanced quality of life. These consistent findings across diverse outcomes support the inclusion of the Mediterranean Diet as a dietary recommendation for individuals with metabolic diseases [47].

Women often face unique challenges due to lower average basal metabolic rate (BMR) compared to men, requiring tailored caloric control to prevent weight gain [80]. Additionally, women typically exhibit a gynoid fat distribution pattern characterized by a greater fat storage in hips and thighs and tend to preserve lean body mass more effectively than men under calorie restriction, possibly due to protective hormonal and metabolic mechanisms [81].

Long-term adherence to balanced dietary patterns such as the Mediterranean Diet rich in whole grains, legumes, fruits, vegetables, lean proteins, and healthy fats has been consistently associated with sustained weight control and reduced risk of obesity and related chronic diseases, including cardiovascular diseases, type 2 diabetes, and certain cancers [82,83]. Energy restriction remains the cornerstone of effective dietary strategies for overweight and obesity management. However, not all low-calorie diets yield equal long-term success in health outcomes. Evidence suggested that a hypocaloric Mediterranean Diet is more effective in sustaining weight over time compared to other dietary models, including low-carbohydrate, low-fat, high-protein diets or intermittent fasting regimens [84,85]. The superior effects of the Mediterranean Diet are attributed not only to its palatability and nutrient density but also to its positive impact on satiety, insulin sensitivity, and inflammation [86,87]. Addressing obesity in women requires personalized approaches that consider biological differences, such as reproductive hormonal fluctuations, psychosocial stressors, caregiving responsibilities, and societal factors. An integrative approach that aligns nutritional quality with individual lifestyle, metabolic needs, and psychosocial context offers the most promising path to effective and sustainable obesity prevention in women.

### 5.3. Cardiovascular Health Considerations

Although the global burden of cardiovascular diseases (CVDs) has steadily declined over the last decade, they remain the leading cause of morbidity and mortality globally, accounting for about 35% of all deaths among women worldwide [88,89]. The development of CVDs is partly due to unmodifiable factors, such as age, sex, and genetic determinants [88].

Sex differences have been observed for some traditional cardiovascular risk factors such as hypertension, type 2 Diabetes Mellitus (DM), and sodium intake [90]. In more detail, a greater susceptibility to the cardiovascular consequences of hypertension has been observed in women, which occurs at lower blood pressure (BP) levels than in men [91]. The incidence of type 2 DM overlaps between men and women; however, complications have been shown to be more severe in women [90]. With regard to sodium intake, women show a greater sodium sensitivity, so high salt intake has a greater impact on increasing BP levels in women than in men [91].

Moreover, there are same additional pregnancy related risk factors: preterm delivery (birth at <37 weeks’ gestation), preeclampsia (new onset hypertension after 20 weeks’ gestation in a woman previously normotensive), gestational DM (newly diagnosed DM beyond the first trimester of pregnancy); these conditions are independent risk factors for the development of future long-term CVDs [92].

Despite this predisposition, a large proportion of the overall CVD burden is attributable to behavioral risk factors (tobacco use, insufficient physical activity, unhealthy diet, and harmful use of alcohol), which makes CVDs partly preventable or controllable through lifestyle interventions [93]. Unhealthy dietary habits contribute most significantly, among all behavioral risk factors, to CVDs mortality risk in Europe [94]. On the contrary, the adoption of a healthy diet, such as the Mediterranean dietary pattern, reduces cardiovascular risk, through its beneficial effects on risk factors such as lipid and glycemic profile, BP, and body weight [95]. More specifically, the role of the Mediterranean Diet in cardiovascular health has been widely documented, with evidence supporting both primary prevention and secondary management [47]. The purpose of this paragraph is to explore the impact of different food components on cardiovascular health and the best dietary strategies to implement in order to control cardiovascular risk factors.

#### 5.3.1. Glycemic Profile

A diet high in dietary fiber has been shown to improve blood glucose control, cholesterol levels, and body weight, thereby supporting diabetes management and reducing the risk of cardiometabolic diseases [96]. The WHO recommends a daily intake of at least 25 g of dietary fiber naturally present in foods [97]. Primary sources of fiber include whole fruits and vegetables, whole grains such as brown rice, whole wheat, rye, oats, and barley, as well as legumes, nuts, and seeds. Importantly, no additional benefits on glycemic control have been observed from the use of fiber supplements compared to fiber-rich foods. Therefore, supplements should only be considered when dietary intake alone is insufficient [96].

Regarding carbohydrates, a wide range of total carbohydrate intake can be acceptable in diabetes management, provided that recommendations concerning dietary fiber, sugars, saturated fats, and protein intake are met [96]. However, extremely low-carbohydrate diets, such as ketogenic diets, are not recommended. Evidence has not demonstrated any clear benefit of such diets in the prevention or management of type 2 diabetes mellitus (DM), and potential safety concerns have been identified, including increased levels of LDL, risk of hypoglycemia, ketoacidosis, and deficiencies in essential vitamins and minerals [96]. Furthermore, it is advisable to limit the intake of free or added sugars to less than 10% of total daily energy intake [98]. Higher levels of sugar consumption have been associated with increased body weight, elevated fasting glucose and insulin levels, higher triacylglycerols and uric acid concentrations, and a greater risk of metabolic syndrome, hypertension, gout, and cardiovascular diseases, particularly in individuals with or at risk for diabetes [96].

The quality of carbohydrates plays a crucial role in preventing the onset of type 2 DM. This is commonly evaluated through the glycemic index (GI) and glycemic load (GL), which measure the rate at which carbohydrate-containing foods are broken down during digestion and their subsequent effect on blood glucose levels. While the GI indicates how quickly blood sugar rises after consuming a specific food, the GL takes portion size into account, providing a more comprehensive picture of its glycemic impact [99]. Following a diet based on low GI or GL foods has been shown to improve glycemic control and reduce intermediate cardiometabolic risk factors in individuals with diabetes [96]. Comprehensive tables listing the GI and GL of specific foods are available for reference in the updated international database [99].

Non-sugar sweeteners (NSS), commonly used as low- or no-calorie substitutes for free sugars, are frequently added to pre-packaged foods and beverages or used directly by consumers. While NSS are often promoted for aiding weight control and are sometimes recommended to help manage blood glucose levels in individuals with diabetes, current evidence does not support long-term benefits on body fat reduction [96,100]. Moreover, potential adverse effects have been identified with prolonged use of NSS, including an increased risk of type 2 DM, cardiovascular diseases, and overall mortality. For these reasons, the WHO advises against the use of NSS as a strategy for weight management or for reducing the risk of noncommunicable diseases in the general population [100].

Lastly, the quantity and quality of dietary fat are also critical factors in diabetes management. Saturated fatty acids (SFAs), in particular, have been shown to negatively affect glycemic control and increase the risk of diabetes. Replacing SFAs with PUFAs or monounsaturated fats (MUFAs) has been associated with reductions in glycated hemoglobin levels, fasting glucose concentration, and improvements in insulin resistance as measured by the Homeostatic Model Assessment (HOMA-IR) [96]. For more detailed strategies on reducing SFA intake, further reference is available in the section discussing lipid profiles.

#### 5.3.2. Lipid Profile

A substantial body of evidence indicates that excessive intake of saturated fatty acids (SFAs) leads to elevated LDL levels, which remain a primary target in cardiovascular risk reduction strategies [95]. Conversely, replacing SFAs isocalorically with MUFAs and PUFAs has demonstrated beneficial effects on cardiovascular health outcomes [101]. For this reason, the WHO recommends limiting SFAs to no more than 10% of total energy intake. It is advised to substitute them with PUFAs and MUFAs, preferably from plant-based sources, or with carbohydrates derived from fiber-rich foods such as whole grains, vegetables, fruits, and pulses [102].

To meet these dietary goals, it is suggested to increase the consumption of PUFA-rich foods, particularly fatty fish like anchovies, sardines, and mackerel at least once a week, along with three times a week intake of nuts (around 30 g). Incorporating monounsaturated fats, especially extra virgin olive oil, and reducing the intake of meat—especially processed meat—and full-fat dairy products like regular cheese and butter, which are high in SFAs, is also recommended [95,101,102]. Additionally, to further lower total cholesterol and LDL-C levels, a dietary cholesterol intake of less than 300 mg per day is advised, particularly for individuals with elevated plasma cholesterol concentrations [102].

Trans fatty acids (TFAs) present another area of concern, given their adverse impact on both LDL-C and HDL-C levels. Elevated intake of TFAs has been associated with a marked increase in coronary heart disease (CHD) incidence and related mortality [95]. WHO guidelines suggested keeping TFA intake to 1% or less of total energy intake, again recommending replacement with PUFAs or MUFAs from plant-based sources [102]. While TFAs occur naturally in small amounts in dairy products and meat from ruminant animals—such as cattle, sheep, goats, and camels—the primary source of dietary TFAs remains industrially produced fats, generated through the partial hydrogenation of oils. These industrial TFAs are commonly found in baked and fried goods, processed snacks, packaged foods, and certain cooking oils and spreads [103]. Although no specific food is strictly prohibited, minimizing the intake of products high in industrial TFAs and fried goods is strongly advised [102].

In terms of dietary carbohydrates, they generally exert a neutral effect on LDL-C. However, excessive carbohydrate consumption, particularly from refined sources, may negatively influence triglyceride (TG) levels and HDL-C concentrations. Similarly, a high intake of dietary fructose has been shown to raise TG levels, especially in individuals with existing hypertriglyceridemia or abdominal obesity. On the other hand, dietary fiber—particularly soluble fiber—has cholesterol-lowering properties. Replacing saturated fats with fiber-rich foods not only optimizes LDL-C levels but also counterbalances the potential negative effects of high-carbohydrate diets on other lipoproteins. To support a healthy lipid profile, a predominantly plant-based diet is recommended, emphasizing foods rich in soluble fiber such as fruits, vegetables, legumes, and whole grains. Carbohydrates should ideally account for 45–55% of total daily energy intake. Moreover, added sugars should be restricted to no more than 10% of total energy, with special caution regarding soft drink consumption, particularly among individuals prone to elevated TG levels or central obesity [101].

Alcohol intake is another modifiable factor linked to cardiovascular health. Alcohol consumption has been associated with an increased risk of several cardiovascular diseases, including various forms of stroke, coronary artery disease (CAD), and heart failure [95]. Specifically, alcohol’s most notable adverse effect on the lipid profile is the elevation of TG levels. Given that the lowest cardiovascular risk is observed in abstainers, refraining from alcohol is preferable. Alcohol consumption during pregnancy is not recommended, as prenatal exposure to alcohol is associated with a wide range of adverse effects on fetal development. For additional details on dietary strategies aimed at improving lipid profiles, refer to Table 1.

#### 5.3.3. Blood Pressure

Excessive sodium consumption has been strongly related to elevated BP levels, both in the general population and among individuals with hypertension. Reducing sodium intake is considered fundamental not only for the prevention of hypertension but also as an effective strategy for managing BP in patients already diagnosed with hypertension, often allowing for a reduction in the need for antihypertensive medications [91]. To lower BP and reduce the risk of CVDs, including stroke and CHD, the WHO recommends limiting sodium intake to less than 2 g per day, equivalent to 5 g of salt daily [104]. Achieving this target involves prioritizing fresh foods naturally low in sodium—such as fruits, vegetables, and legumes—while minimizing consumption of processed foods like savory snacks, sauces, spreads, and processed meats, which tend to be high in sodium content [103,104].

In contrast, potassium intake plays a protective role in cardiovascular health. Insufficient potassium consumption has been associated with an increased risk of various non-communicable diseases (NCDs), including CVDs, kidney stone formation, and reduced bone mineral density [105]. Adequate potassium intake has been shown to effectively lower both systolic and diastolic blood pressure levels. Reflecting this, WHO recommends increasing potassium intake to at least 3510 mg per day, which has been shown to significantly reduce BP and the risk of CVDs, stroke, and CHD [105]. Potassium-rich foods include fruits and vegetables, low-fat dairy products, selected fish and meats, nuts, and soy-based products. Consuming four to five servings of fruits and vegetables daily alone can provide between 1550 and over 3000 mg of potassium [91].

Alcohol intake is another key factor influencing blood pressure. Several studies have demonstrated a strong positive linear association between alcohol consumption and increased BP levels. Therefore, a significant reduction in alcohol intake—ideally approaching abstinence—is recommended for both adult men and women to support blood pressure management and overall cardiovascular health [91].

### 5.4. Diet and Bone Health

#### 5.4.1. Factors Influencing Bone Mass

Bone mass fluctuates throughout life, generally increasing slowly and accelerating during adolescence to reach a peak in the early third decade. Subsequently, bone mass declines in correlation with aging and the onset of diseases [106]. While bone accumulation is predominantly governed by genetic factors, such as the vitamin D receptor (VDR), collagen 1α1 (COL1A1), IL-6, IL-10, and TGF-β1, these hereditary influences are highly polygenic and account for approximately 50–80% of the variance in bone mass. Conversely, modifiable and environmental factors, particularly nutrition, play a significant role in bone health [107,108,109].

Nutrition is a critical factor influencing bone mass, with the potential to either increase or decrease it. Numerous studies demonstrate that nutritional supplementation with vitamin E, vitamin D, calcium, or long-chain omega-3 polyunsaturated fatty acids is linked to increased bone mass [110]. An observational study found that the total antioxidant capacity of the diet is positively associated with bone mineral density (BMD) in the lumbar spine and femoral neck of premenopausal women, and it also reduces the risk of osteoporosis in postmenopausal women [111]. The evidence base for the Mediterranean Diet in the context of musculoskeletal disorders remains limited but promising. For primary prevention, observational data suggest that adherence to the Mediterranean Diet may contribute to a reduced incidence of fractures, supporting its role as a beneficial lifestyle strategy for bone health. In patients already suffering from musculoskeletal conditions, only one cohort study has been identified, focusing on individuals with osteoarthritis. The results indicated that higher adherence to the Mediterranean Diet was associated with a modest yet statistically significant reduction in the risk of pain, with a moderate level of certainty. Although the current evidence is scarce, these findings open an important avenue for future research and suggest that the Mediterranean Diet may play a role in both prevention and symptom management of musculoskeletal diseases [47].

#### 5.4.2. Bone Health in Women of Childbearing Age

In women of childbearing age, physiological reductions in bone mass during pregnancy and lactation can lead, depending on nutritional status, to conditions such as pregnancy and lactation-associated osteoporosis (PLO) [112]. During pregnancy, there is an increased demand for calcium as it is transferred from the maternal circulation to the developing fetus to ensure proper mineralization of the fetal skeleton. This process is accompanied by heightened bone turnover and is regulated by hormonal factors that enhance calcium and phosphorus absorption in the maternal intestine. Specifically, levels of dihydroxyvitamin D, placental lactogen, prolactin, estradiol, and parathyroid hormone-related protein (PTHrP) increase [106]. However, the mobilization of calcium from the maternal skeleton is more pronounced in women with inadequate dietary calcium intake or deficiencies in factors crucial for calcium absorption, such as vitamin D [113].

Lactation similarly prompts changes in bone metabolism to support the growth of the newborn, characterized by reduced calcium excretion through breast milk. Additionally, the suppression of the hypothalamic-pituitary-ovarian axis leads to a marked decrease in estrogen levels, resulting in increased bone turnover. Consequently, there is a notable decrease in BMD during and after lactation, which varies across different skeletal regions [106].

The mother’s diet and nutritional intake during pregnancy and breastfeeding are crucial not only for her bone health but also for the bone development of the fetus and child during early years [114]. A multicentric, randomized, double-blind, placebo-controlled study, known as the Maternal Vitamin D Osteoporosis Study (MAVIDOS), demonstrated that vitamin D supplementation in pregnant women with low initial levels increases neonatal bone mass, particularly for those born in winter, and enhances areal bone mineralization (aBMD) by age four, irrespective of birth season. Additionally, stronger mother-child interactions were noted, particularly among children with low milk intake and physical activity, highlighting the potential of recovery through adequate nutrition. These findings suggested a lasting positive effect of maternal vitamin D supplementation during pregnancy on children’s bone health at age four, although further research is needed [113]. This influence may be attributed to maternal vitamin D insufficiency, which can alter placental calcium transport via changes in PTHrP [115].

Furthermore, the intake of nutrients such as magnesium, phosphorus, potassium, and folate positively correlates with maternal BMD and supports bone development during pregnancy, childhood, and puberty in offspring [116,117]. Among micronutrients, iron is important for maintaining bone health; both deficiency and excess can harm bone health through various mechanisms. Children and pregnant women are particularly susceptible to iron deficiency, with approximately 40% of pregnant women and 30% of non-pregnant women affected [118]. Although the relationship between iron deficiency and bone density is complex, a positive correlation exists between dietary iron intake and bone density in women, contingent upon sufficient calcium intake [119].

#### 5.4.3. Mediterranean Diet and Bone Health

From a nutritional standpoint, the Mediterranean diet is an exemplary model for supporting bone health, including during pregnancy and lactation. This diet emphasizes a high consumption of vegetables, legumes, fruits, and cereals; it favors fish over meat and dairy products for protein, prioritizes unsaturated fats from olive oil over saturated fats, and includes moderate wine consumption. The nutritional profile of the Mediterranean diet is rich in natural bioactive molecules with antioxidant, anti-inflammatory, and alkalizing properties, alongside essential nutrients like calcium and vitamins that enhance bone health.

Particularly noteworthy are plant-derived phytochemicals such as polyphenols (including flavonoids and stilbenes) and non-polyphenolic compounds like terpenoids. These compounds not only combat bone-wasting diseases like osteoporosis and osteoarthritis but also regulate important processes like osteogenesis, chondrogenesis, and osteoclastogenesis, which are crucial for maintaining the skeletal system [107,120].

Evidence from a substantial clinical study underscores the protective effects of specific vegetables on bone health. Conducted over 14.5 years, the study identified that higher consumption of cruciferous and allium vegetables correlated with a reduced risk of fractures in women, highlighting the beneficial properties of organosulfur compounds prevalent in these vegetables [121,122].

Regarding ethanol, while generally considered detrimental to bone health, wine consumption presents an exception due to its rich content of bioactive compounds such as resveratrol. These elements promote osteogenesis and inhibit osteoclastogenesis, indicating that the positive effects of wine on bone health may stem from these compounds rather than ethanol per se [123].

Additionally, fish provide essential minerals and vitamins, including selenium, calcium, iodine, zinc, and vitamins A and D, crucial for bone health and optimal mineralization [107,115,124].

## 6. Diet and Reproductive Health

### 6.1. Influence of Diet on Menstrual Cycle

The menstrual cycle is a vital indicator of reproductive health, intricately regulated by a dynamic interplay of hormones. Diet is a major influencer of its regularity and function, playing a significant role in the stability of the hypothalamic-pituitary-ovarian (HPO) axis. When diet is inadequate or imbalanced, it can disrupt this hormonal axis, resulting in irregular menstrual cycles or even amenorrhea [125].

Severe caloric deficits or poor dietary intake can lead to hypothalamic amenorrhea, where the hypothalamus reduces the release of gonadotropin-releasing hormone (GnRH), effectively halting follicular development and ovulation. Research has shown that a deficiency in energy availability results in disruptions to GnRH, thus causing menstrual disturbances like amenorrhea [126]. This is commonly seen in athletes or individuals with extremely restricted diets. Indeed, extreme caloric restriction has been shown to affect menstrual regularity in female athletes, making it crucial to maintain adequate caloric intake for menstrual health, especially for those involved in high levels of physical activity [127].

On the other end of the spectrum, over-nutrition and excessive body weight can also cause significant reproductive disruptions. Excess adipose tissue increases estrogen production through the process of aromatization, which converts androgens into estrogens. Specifically, estrone (E1) a weaker estrogen than estradiol (E2) is synthesized in the adipose tissue. High levels of less active estrogen, can interfere with the HPO axis and contribute to conditions such as anovulation and PCOS. Studies have illustrated that obesity contributes to reproductive disorders by elevating estrogen and other hormonal imbalances that disrupt ovulatory cycles, emphasizing the link between obesity, hormonal dysregulation, and infertility outcomes in women of reproductive age [128,129].

Micronutrients are equally crucial in maintaining menstrual regularity. Vitamin D and iron deficiencies have been linked to menstrual disorders and increased menstrual pain [130]. Similarly, deficiencies in iron can lead to anemia, which impacts blood flow and overall ovarian function, leading to cycle irregularities and fatigue, which further disrupts hormonal balance. The importance of minerals—such as calcium, magnesium, zinc, and selenium—has been suggested in supporting various phases of the menstrual cycle, particularly in regulating ovulation and ensuring endometrial health [131].

A diet rich in whole foods, including leafy greens, healthy fats, plant-based proteins, and fortified cereals, can help maintain hormonal harmony necessary for a healthy menstrual cycle. It is essential to avoid severe calorie restriction or over-consumption of processed foods to support the HPO axis’s proper function.

### 6.2. Hormonal Regulation and Fertility

Hormones govern every stage of the reproductive process, from ovulation to embryo implantation. Nutritional status profoundly influences both the production of these hormones and their sensitivity in the body, affecting overall fertility [132].

The balance of estrogen and progesterone is pivotal for fertility. Diets high in trans fats and refined carbohydrates can contribute to insulin resistance, which indirectly impacts ovarian function. Insulin resistance can interfere with the release of FSH and LH, leading to poor follicular development and anovulation [133]. In contrast, n-3 PUFAs, found in fatty fish and flaxseeds, promote the synthesis and balance of these sex hormones. Moreover, diets rich in healthy fats could positively influence ovulatory function by reducing inflammation and supporting ovarian follicle development [134].

Insulin resistance is a hallmark of PCOS, a condition that often leads to anovulatory infertility. PCOS patients benefit significantly from dietary strategies that enhance insulin sensitivity, such as low glycaemic index diets and careful weight management. Reducing insulin resistance through diet improves ovulation rates and overall fertility in women with PCOS. Including fiber-rich carbohydrates and healthy fats in meals, while avoiding simple sugars and refined carbs, can help stabilize insulin levels, thereby improving reproductive outcomes [135].

Energy homeostasis is mediated by hormones like leptin and ghrelin, both of which are highly influenced by nutrition. Leptin is produced by fat cells and plays a role in regulating hunger and energy expenditure, while ghrelin stimulates appetite. Extreme diets and low body fat levels can reduce leptin production, which in turn disrupts the communication with the hypothalamus, potentially leading to reproductive dysfunction. Both leptin deficiency and excess cause disturbances in ovulation and menstrual cycle regulation.

Managing hormonal balance through diet, such as incorporating balanced healthy fats and avoiding processed foods, can help optimize fertility and prepare the body for conception [135,136].

### 6.3. Nutrients Essential for Promoting Fertility

Certain nutrients are absolutely essential for maintaining reproductive health and enhancing fertility, due to their crucial roles in ovulation, hormone production, and early embryonic development. These nutrients contribute significantly to optimizing reproductive potential by ensuring the body is adequately supported in its reproductive functions [132].

Folate is one of the key nutrients in reproductive health. It is important for DNA synthesis and cell division, making it vital for the formation and proper development of the embryo. Folate is especially important in the early stages of pregnancy, as it helps reduce the risk of neural tube defects in the developing fetus [137].

A diet rich in folate supports overall fertility and is linked with successful pregnancy outcomes. Foods high in folate include leafy greens such as spinach and kale, legumes like lentils and beans, as well as fortified cereals. Adequate folate intake is important for reproductive health, enhancing ovulatory function and reducing the risk of developmental issues during pregnancy [44]. Thus, ensuring an adequate intake of folate is essential for all women of reproductive age, particularly for those attempting to conceive, as it lays a foundational support for a healthy pregnancy [138].

Iron is another vital nutrient necessary for female fertility. Iron deficiency can negatively affect ovulation, leading to irregular menstrual cycles or anovulation, which can decrease fertility potential. Both heme iron—found in animal products such as red meat and poultry—and non-heme iron—found in plant sources such as legumes, spinach, and fortified cereals—are important for maintaining optimal iron levels. Consuming a combination of these iron sources ensures that the body receives sufficient amounts to support ovulatory function and overall reproductive health. Moreover, pairing non-heme iron sources with foods rich in vitamin C—such as citrus fruits, bell peppers, and strawberries—significantly enhances iron absorption [131,139]. Ensuring sufficient iron intake is associated with improved fertility outcomes, highlighting the importance of iron-rich foods in preconception nutrition [140].

Zinc is also indispensable for reproductive health, playing an integral role in ovulation, enzyme activation, and cell division. For both men and women, zinc is necessary to ensure proper reproductive function. In women, zinc supports follicle development, oocyte maturation, and hormonal balance, all of which are crucial for ovulation. In men, zinc is essential for sperm production and quality, including motility and structural integrity. Zinc deficiency has been linked to disruptions in the levels of reproductive hormones, which can impair ovulation and lead to reduced egg quality, thereby affecting fertility [32]. Foods such as nuts, seeds, legumes, and seafood—as oysters, which are particularly high in zinc—are excellent dietary sources that help maintain optimal zinc levels and support reproductive function [32].

n-3 PUFAs are well-known for their anti-inflammatory properties, which make them especially beneficial for enhancing fertility. Found abundantly in fatty fish like salmon and mackerel, as well as plant-based sources such as flaxseeds, chia seeds, and walnuts, omega-3 fatty acids contribute to improved ovarian function, reduced inflammation, and enhanced embryo implantation. Chronic inflammation is a factor that can disrupt the reproductive process, impacting ovulation and the receptivity of the endometrium. Omega-3s counteract inflammation, creating a healthier environment for ovulation and embryo development. Moreover, these essential fatty acids improve blood flow to the reproductive organs, optimizing the conditions needed for conception. Therefore, the inclusion of n-3 PUFAs in the diet is key for individuals aiming to boost their fertility potential and enhance the chances of successful conception [21].

Antioxidants, including vitamins C and E, and selenium play a pivotal role in protecting the reproductive system from oxidative stress. Oxidative stress can harm oocytes and embryos, affecting their viability and quality, which in turn reduces fertility. Antioxidants help neutralize free radicals, thereby protecting cells, supporting a healthy reproductive environment, and reducing damage that can impair fertility [141]. Vitamin C, which can be found in citrus fruits, berries, and bell peppers, works synergistically with vitamin E (found in extra virgin olive oil, nuts, pistachios) to enhance the antioxidant defense system, while selenium, found in nuts, fish, and whole grains, also plays a crucial role in supporting antioxidant function and maintaining hormonal balance. Thus, antioxidants play a vital role in reproductive health by creating an environment conducive to fertilization and implantation. They help combat oxidative stress, which can damage eggs and sperm, thereby improving their quality and viability. Incorporating antioxidant-rich foods, such as fruits, vegetables, nuts, and seeds, into the diet can significantly enhance egg quality and overall reproductive outcomes, supporting successful conception and pregnancy [52].

A nutrient-dense diet that includes a variety of these essential nutrients—folate, iron, zinc, n-3 PUFAs, and antioxidants—can significantly boost fertility potential. The combination of these nutrients supports a balanced hormonal environment, improved egg quality, efficient ovulation, and a receptive endometrium, all of which are critical factors for successful conception and pregnancy. By focusing on a well-rounded diet that incorporates a wide range of whole foods, individuals seeking to optimize their fertility can benefit from improved reproductive health, reduced risk of pregnancy complications, and enhanced overall well-being in their reproductive journey.

### 6.4. Dietary Strategies to Optimize Reproductive Health

Adopting specific dietary strategies can significantly enhance overall reproductive health, leading to improved fertility outcomes for women trying to conceive. The role of diet in fertility is multifaceted, influencing hormone production, ovulation, and even early embryonic development. By incorporating certain dietary patterns and avoiding harmful food components, women can create a nutritional environment that promotes hormonal harmony and reproductive function.

The Mediterranean diet is one of the most highly recommended dietary patterns for improving reproductive health. Rich in whole grains, fruits, vegetables, nuts, seeds, and olive oil, this diet is characterized by its emphasis on plant-based foods, healthy fats, and antioxidants, making it a powerful approach to support fertility. Research highlighted positive associations between adherence to the Mediterranean diet and increased rates of successful pregnancies, particularly in assisted reproductive treatments [142]. The components of the Mediterranean diet, such as n-3 PUFAs, vitamins, and polyphenols, work together to reduce inflammation, enhance blood flow to reproductive organs, and maintain optimal hormonal levels. This dietary pattern has also been linked to a reduced risk of miscarriage, partly attributed to its positive effects on metabolic health, improved insulin sensitivity, and reduced oxidative stress—all of which play critical roles in conception and sustaining early pregnancy. By promoting cardiovascular health and reducing metabolic disturbances, the Mediterranean diet helps create a supportive environment for ovulation and embryo implantation, making it an effective dietary strategy for enhancing fertility outcomes [143].

Another crucial dietary strategy for enhancing reproductive health is avoiding trans fats. Trans fats, which are commonly found in processed foods, margarine, and commercially baked and fried goods, have been shown to negatively impact insulin sensitivity and ovarian function. These harmful fats can disrupt cell membrane integrity and impair the functionality of reproductive tissues, ultimately affecting ovulation and hormone production. Additionally, trans fats contribute to systemic inflammation, which further impacts reproductive health negatively [144]. To enhance fertility outcomes, it is essential to minimize the intake of trans fats by choosing healthier fat sources, such as those found in nuts, seeds, avocados, and olive oil. By avoiding trans fats and incorporating healthier dietary fats, women can improve hormonal regulation, maintain better insulin sensitivity, and reduce inflammation—all of which are crucial for reproductive success.

Maintaining balanced macronutrient intake—ensuring appropriate ratios of carbohydrates, proteins, and fats—is critical for reproductive health. A balanced macronutrient intake helps maintain metabolic stability, regulate insulin levels, and support hormonal production, all of which are essential for a healthy menstrual cycle and ovulation. Carbohydrates provide the necessary energy for cellular functions, while proteins contribute to the repair and formation of reproductive tissues. Healthy fats are involved in the synthesis of sex hormones such as estrogen and progesterone. In this context, a balanced diet should consist of complex carbohydrates (such as whole grains and vegetables), lean proteins (such as legumes, fish, poultry and skimmed dairy products), and healthy fats (like those found in olive oil, nuts, and avocados). This macronutrient balance ensures that the reproductive system functions smoothly, supporting regular cycles and providing the body with the necessary building blocks for optimal fertility [132].

Hydration and lifestyle choices are also essential aspects of reproductive health. Adequate hydration is fundamental to maintaining healthy cellular functions, ensuring that nutrients are effectively transported throughout the body and that waste products are efficiently removed. Proper hydration also supports the production of cervical mucus, which is necessary for sperm mobility and successful fertilization. In addition to hydration, it is important to limit the intake of caffeine and alcohol. Excessive caffeine consumption has been linked to delayed conception and an increased risk of miscarriage, while alcohol can interfere with hormone regulation and negatively impact ovarian function [145]. The importance of lifestyle modifications, including diet, was emphasized as an effective measure to improve reproductive health outcomes. Incorporating mindfulness practices, stress management, regular exercise, and avoiding smoking are all lifestyle changes that, alongside a balanced diet, can enhance fertility. Managing stress can help regulate cortisol levels, which in turn supports more regular ovulation and hormonal balance [135].

Tailoring dietary approaches to individual needs is crucial for ensuring long-term reproductive health and optimizing fertility. Consulting with a nutritionist or healthcare provider can help individuals create personalized nutrition plans that address their unique dietary needs and reproductive goals. For women attempting to conceive, personalized advice may include adjusting nutrient levels, managing weight, and addressing any specific deficiencies or metabolic concerns that might hinder conception. For example, a woman with PCOS may need a diet specifically tailored to enhance insulin sensitivity and manage symptoms through low-glycemic index foods and consistent meal planning.

Integrating balanced nutrition into daily life can significantly enhance menstrual health, promote hormonal balance, and improve overall fertility. A well-rounded diet that includes whole grains, healthy fats, plant-based proteins, and minimizes ultra-processed foods sets a strong foundation for optimal reproductive health. It also helps prepare the body for conception, reduces the risk of miscarriage, and supports a healthy pregnancy. These dietary strategies thus pave the way for healthier outcomes throughout the entire reproductive journey, supporting not only conception but also early fetal development and successful gestation.

## 7. Discussion

**Figure 1:**
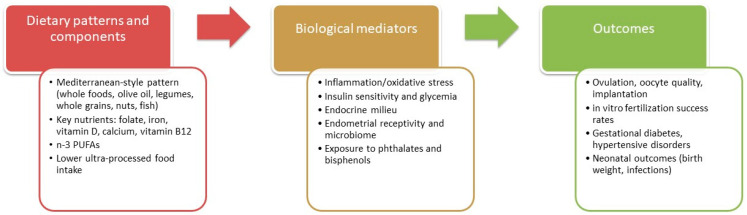
Diet-mediator-outcome pathways in female reproductive and pregnancy health. This figure is based on [41,43,44,54,65,70,72].

This position paper highlights the central role of nutrition in protecting and improving the health of women of childbearing age—a group whose well-being shapes their own health trajectories, reproductive outcomes, and the health of future generations. The evidence aligns with the diet-mediator-outcome framework in Figure 1, in which diet quality and key nutrients act through inflammatory, metabolic, and endocrine pathways to influence reproductive and pregnancy outcomes.

Consistent with our consensus-based narrative approach, we did not follow formal systematic-review procedures (protocol registration, exhaustive multi-database searches, dual independent screening and data extraction, PRISMA flow reporting, or quantitative synthesis/meta-analysis). This manuscript is therefore a position paper that synthesizes and interprets evidence to inform public-health guidance. Across the body of evidence, one message is clear: adequate macro- and micronutrient intake is essential to meet the physiological demands of menstruation, fertility, pregnancy, lactation, and long-term metabolic health. For translation into practice, Table 2 summarizes evidence-informed dietary recommendations for women of childbearing age.

In particular, a diversified, balanced pattern rich in whole and minimally processed foods is associated with better hormonal regulation, improved reproductive outcomes, optimal fetal development, and a lower risk of conditions such as anemia, gestational diabetes mellitus (GDM), and preeclampsia. Special emphasis is placed on the Mediterranean dietary pattern, whose high content of fiber, antioxidants, unsaturated fats, and essential micronutrients has consistently been linked with favorable metabolic profiles, ovulatory function, and pregnancy outcomes.

Conversely, mounting data warns against the overconsumption of ultra-processed foods, whose high content of added sugars, trans fats, and sodium is associated with systemic inflammation, insulin resistance, obesity, and adverse reproductive outcomes. Likewise, the strategic use of dietary supplements emerges as critical, particularly for nutrients such as folate, vitamin D, iron, and DHA, which may be insufficient in typical diets or in vulnerable populations. However, supplementation must be personalized, carefully dosed, and supervised, avoiding the pitfalls of unnecessary excesses.

These findings have important implications for public health strategies. It is essential that nutritional policies specifically tailored to the needs of women of reproductive age become central to health agendas. Education campaigns should be reinforced, not only to raise awareness about healthy eating patterns but also to empower women with knowledge that enables informed dietary choices, particularly during preconception and pregnancy periods. Integrating individualized nutrition counseling into routine healthcare services—including gynecological visits, family planning programs, and prenatal care—should be a priority.

Moreover, to address structural inequalities, interventions must focus on making nutrient-dense, affordable foods more accessible, especially in disadvantaged or low-resource communities where food insecurity heightens the risk of micronutrient deficiencies. Public policies promoting local food systems, regulating misleading marketing of ultra-processed foods, and encouraging fortification programs could play a transformative role in improving overall dietary quality.

However, an additional layer of complexity demands attention: the necessity of personalization. Nutritional needs can vary considerably based on factors such as age, socioeconomic status, cultural context, underlying medical conditions, lifestyle, and personal preferences. Public health recommendations, while rooted in general scientific principles, should increasingly embrace a more individualized approach, recognizing that one-size-fits-all solutions may fail to address specific vulnerabilities or challenges faced by subgroups within this population. Tailored interventions, supported by trained healthcare professionals, can better cater to diverse needs, enhancing adherence and long-term effectiveness.

Finally, while the current body of evidence is substantial, there remains an ongoing need for further research. Longitudinal studies that explore the long-term impact of dietary patterns starting from preconception through postpartum are essential. Additionally, more data are needed to clarify the complex interactions between diet, genetics, the gut microbiome, hormonal profiles, and environmental exposures in determining reproductive and metabolic outcomes in women of childbearing age.

## 8. Conclusions

In conclusion, prioritizing optimal nutrition for women during their reproductive years is a critical investment not only in individual health and reproductive success but also in the well-being of future generations and the broader public health landscape. Policymakers, healthcare providers, researchers, and communities alike must collaborate to implement evidence-based, accessible, and culturally sensitive nutritional strategies that empower women and foster healthier societies. Only through a concerted, personalized, and research-informed approach can we fully harness the potential of nutrition as a cornerstone of reproductive and intergenerational health.

## Figures and Tables

**Table 1:** Food choices to lower LDL-C and improve the overall lipoprotein profile [101].

	To Be Preferred	To Be Used in Moderation	To Be Chosen Occasionally Amounts
Cereals	Whole grains	Refined bread and pasta, biscuits, corn flakes	Pastries, muffins, pies, croissants
Vegetables	Raw and cooked vegetables	Potatoes	Vegetables prepared in butter or cream
Legumes	Lentils, beans, fava beans, peas, chickpeas, soybean		
Fruit	Fresh or frozen fruit	Dried fruit, jelly, jam, canned fruit, sorbets, ice lollies/popsicles, fruit juice	
Sweets and sweeteners		Sucrose, honey, chocolate	Cakes, ice creams, soft drinks, sweets/candies
Meat and fish	Lean and oily fish, poultry without skin	Lean cuts of beef, lamb, pork, and veal, seafood, shellfish	Sausages, salami, bacon, spare ribs, hot dogs, organ meats
Dairy food and eggs	Skimmed milk and yogurt	Low-fat milk, low-fat cheese and other milk products, eggs	Regular cheese, cream, whole milk and yogurt
Cooking fat and dressing	Vinegar, mustard, Olive oil	non-tropical vegetable oils, soft margarines, salad dressing, mayonnaise, ketchup	Trans fats and hard margarines (better to avoid them), palm and coconut oils, butter, lard, bacon fat
Nuts/seeds		All, unsalted (except coconut)	Coconut
Cooking procedures	Grilling, boiling, steaming	Stir-frying, roasting	Frying

**Table 2:** Summary of dietary recommendations for women of childbearing age.

Domain	Recommendation	Typical Targets	Rationale/Evidence
Dietary pattern	Mediterranean-style, minimally processed diet	Vegetables, fruits, legumes, whole grains, nuts; extra-virgin olive oil; fish 2–3/week	Improved fertility/IVF outcomes; better metabolic profiles; lower GDM; better neonatal outcomes [18,41,42,43,44,45]
Diet quality	Achieve dietary diversity (MDD—W ≥ 5 groups/day)	≥5 of 10 FAO food groups/day	Higher likelihood of micronutrient adequacy and better reproductive outcomes [2,3,4,5,6,7]
UPFs	Reduce UPFs (NOVA)	Limit refined carbohydrates, added sugars, saturated/trans fats, sodium	Associations with adverse pregnancy outcomes and inflammation [53,54,55,56,57,58]
Folate	Supplement peri—conceptionally	400 μg/day (higher if clinically indicated)	Prevents NTDs [5,26,35,36,50,116]
Iron	Ensure adequate intake; supplement per status	18 mg/day (non—pregnant); 30–60 mg/day in pregnancy if indicated	Reduces anemia and adverse pregnancy outcomes [23,24,25,26,27,28,29]
Vitamin D	Assess and replete if low	≈600 IU/day; dose to 25(OH)D status	Linked to preeclampsia risk and maternal–fetal outcomes [5,27,28,29,30,31]
Calcium	Meet daily intake; separate from iron	1000–1200 mg/day	Bone health; synergy with vitamin D [5,27,28,29,30,31]
Vitamin B12	Ensure adequacy (esp. vegetarian/vegan)	2.4 μg/day (non—pregnant); 2.6 μg/day in pregnancy	Prevents anemia and neurologic complications [32,33,41]
n-3 PUFAs (DHA/EPA)	Encourage intake; consider DHA supplement	DHA ≥ 200–300 mg/day during conception/pregnancy	Benefits for implantation and offspring neurodevelopment [18,19,20,21,35,36]

Abbreviations: UPF(s) = ultra-processed foods; NOVA = classification of food processing levels; MDD—W = Minimum Dietary Diversity for Women (FAO indicator); FAO = Food and Agriculture Organization; GDM = gestational diabetes mellitus; NTDs = neural tube defects; n-3 PUFAs = omega—3 polyunsaturated fatty acids; DHA = docosahexaenoic acid; EPA = eicosapentaenoic acid; 25(OH)D = 25—hydroxyvitamin D; IVF = in vitro fertilization.

## Data Availability

Not applicable.
